# Supplementary material for: Heat adaptation of phage T7 under an extended genetic code
Source: Virus Evol. 2021 Dec 1;7(2):veab100. doi: 10.1093/ve/veab100 (PMC8923235; doi:10.1093/ve/veab100)
Supplement: veab100_Supp [file veab100_supp.zip › Sequencing_Analysis.zip!/Sequencing_Analysis/A3/evidence/JC_63.html]

BRESEQ :: Evidence


| New junction evidence | | | | | | | | | | | |
| --- | --- | --- | --- | --- | --- | --- | --- | --- | --- | --- | --- |
|  | | seq id | position | reads (cov) | reads (cov) | score | skew | freq | annotation | gene | product |
| \* | ? | NC\_001604.1 | = 7781 | 1662 (0.950) | 68 (0.040) | 53/558 | 20.4 | 4.1% | intergenic (–/–) | *–/–* | –/– |
|  | ? | NC\_001604.1 | 7899 = | 1673 (1.030) |  |  |  |  | intergenic (–/–) | *–/–* | –/– |
| Rejected: Coverage evenness skew score above cutoff. | | | | | | | | | | | |
| Rejected: Frequency below/above cutoff threshold. | | | | | | | | | | | |

Only 100 of 474 total aligned reads displayed.  

`TAGCTACATGGAGGAAACACCTGATGGCTCTTTGCGGCACCCATCGTTCGTAATGTTCCGTGGCACCGAGGACAACCCTCAAGAGAAAATGTAATCACACTGGCTCACCTTCGGGTGGGCCTTTCTGCGTTTATAAGGAGACACTTTATGTTTAAGAAGGTTGGTAAATTCCTTGCGGCTTTGGCAGCTATCCTGACGCTTGCGTATATTCTTGCGGTATACCCTCAAGTAGCACTAGTAGTAGTTGGCGCTTGTTACTTAGCGGCAGTGTGTGCTTGCGTGTGGAGTATAGTTAACTGGTAATACGACTCACTAAAGGAG‑‑‑‑‑‑‑‑‑‑‑‑‑‑‑‑‑‑‑‑‑‑‑‑‑‑‑‑‑‑‑‑‑‑‑‑‑‑‑‑‑‑‑‑‑‑‑‑‑‑‑‑‑‑‑‑‑‑‑‑‑‑‑‑‑‑‑‑‑‑‑‑‑‑‑‑‑‑‑‑‑‑‑‑‑‑‑‑‑‑‑‑‑‑‑‑‑‑‑‑‑‑‑‑‑‑‑‑‑‑‑‑‑‑‑‑‑‑‑‑‑‑‑‑‑‑‑‑‑‑‑‑‑‑‑‑‑‑‑‑‑‑‑‑‑‑‑‑‑‑‑‑‑‑‑‑‑‑‑‑‑‑‑‑‑‑‑‑‑‑‑‑‑‑‑‑‑‑‑‑‑‑‑‑‑‑‑‑‑‑‑‑‑‑‑‑‑‑‑‑‑‑‑‑‑‑‑‑‑‑‑‑‑‑‑‑‑‑‑‑‑‑‑‑‑‑‑‑‑‑‑‑‑‑‑‑‑‑‑‑‑‑‑‑‑‑‑‑‑‑‑‑‑‑‑‑‑‑‑‑‑‑‑‑‑‑‑‑‑‑‑‑‑‑‑‑‑‑‑‑‑‑‑‑‑‑‑‑‑‑‑‑‑‑‑‑‑‑‑‑‑  >  NC_001604.1/7461‑7781`  
`‑‑‑‑‑‑‑‑‑‑‑‑‑‑‑‑‑‑‑‑‑‑‑‑‑‑‑‑‑‑‑‑‑‑‑‑‑‑‑‑‑‑‑‑‑‑‑‑‑‑‑‑‑‑‑‑‑‑‑‑‑‑‑‑‑‑‑‑‑‑‑‑‑‑‑‑‑‑‑‑‑‑‑‑‑‑‑‑‑‑‑‑‑‑‑‑‑‑‑‑‑‑‑‑‑‑‑‑‑‑‑‑‑‑‑‑‑‑‑‑‑‑‑‑‑‑‑‑‑‑‑‑‑‑‑‑‑‑‑‑‑‑‑‑‑‑‑‑‑‑‑‑‑‑‑‑‑‑‑‑‑‑‑‑‑‑‑‑‑‑‑‑‑‑‑‑‑‑‑‑‑‑‑‑‑‑‑‑‑‑‑‑‑‑‑‑‑‑‑‑‑‑‑‑‑‑‑‑‑‑‑‑‑‑‑‑‑‑‑‑‑‑‑‑‑‑‑‑‑‑‑‑‑‑‑‑‑‑‑‑‑‑‑‑‑‑‑‑‑‑‑‑‑‑‑‑‑‑‑‑‑‑‑‑‑‑‑‑‑‑‑‑‑‑‑‑‑‑‑‑‑‑‑‑‑‑‑‑‑‑‑‑‑‑‑‑‑‑‑‑taatacgactcactaaaggagACACTATATGTTTCGACTTCATTACAACAAAAGCGTTAAGAATTTCACGGTTCGCCGTGCTGACCGTTCAATCGTATGTGCGAGCGAGCGCCGAGCTAAGATACCTCTTATTGGTAACACAGTTCCTTTGGCACCGAGCGTCCACATCATTATCACCCGTGGTGACTTTGAGAAAGCAATAGACAAG‑AAACGTCCGGTTCTTAGTGTGGCAGTGACCCGCTTCCCGTTCGTCCGTCTGTTACTCAAACGAATCAAGGAGGTGTTCTGATGGGACTGTTAGATGGTGAAGCCTGGGAAAAA  >  NC_001604.1/7899‑8198`  

                                                                                                                                                                                                                                                                                                                                                                                                                                                                                                                                                                                                                                                
  `GCTACATGGAGGAAACACCTGATGGCTCTTTGCGGCACCCATCGTTCGTAATGTTCCGTGGCACCGAGGACAACCCTCAAGAGAAAATGTAATCACACTGGCTCACCTTCGGGTGGGCCTTTCTGCGTTTATAAGGAGACACTTTATGTTTAAGAAGGTTGGTAAATTCCTTGCGGCTTTGGCAGCTATCCTGACGCTTGCGTATATTCTTGCGGTATACCCTCAAGTAGCACTAGTAGTAGTTGGCGCTTGTTACTTAGCGGCATTGTGTGCTTGCGTGTGGAGTATAGTTAACTGGTAA                                                                                                                                                                                                                                                                                                                                 >  1:216082/1‑301`  
  `GCTACATGGAGGAAACACCTGATGGCTCTTTGCGGCACCCATCGTTCGTAATGTTCCGTGGCACCGAGGACAACCCTCAAGAGAAAATGTAATCACACTGGCTCACCTTCGGGTGGGCCTTTCTGCGTTTATAAGGAGACACTTTATGTTTAAGAAGGTTGGTAAATTCCTTGCGGCTTTGGCAGCTATCCTGACGCTTGCGTATATTCTTGCGGTATACCCTCAAGTAGCACTAGTAGTAGTTGGCGCTTGTTACTTAGCGGCAGTGTGTGCTTGCGTGTGGAGTATAGTTAACTGGTct                                                                                                                                                                                                                                                                                                                                 >  1:266332/1‑299`  
  `GCTACATGGAGGAAACACCTGATGGCTCTTTGCGGCACCCATCGTTCGTAATGTTCCGTGGCACCGAGGACAACCCTCAAGAGAAAATGTAATCACACTGGCTCACCTTCGGGTGGGCCTTTCTGCGTTTATAAGGAGACACTTTATGTTTAAGAAGGTTGGTAAATTCCTTGCGGCTTTGGCAGCTATCCTGACGCTTGCGTATATTCTTGCGGTATACCCTCAAGTAGCACTAGTAGTAGTTGGCGCTTGTTACTTAGCGGCAGTGTGTGCTTGCGTGTGGAGTATAGTTAACTGGTAA                                                                                                                                                                                                                                                                                                                                 <  1:287904/301‑1`  
  `GCTACATGGAGGAAACACCTGATGGCTCTTTGCGGCACCCATCGTTCGTAATGTTCCATGGCACCGAGGACAACCCTCAAGATAAAATGTAATCACACTGGCTCACCTTCGGGTGGGCCTTTCTGCGTTTATAAGGAGACACTTTATGTTTAAGAAGGTTGGTAAATTCCTTGCGGCTTTGGCAGCTATCCTGACGCTTGCGTATATTCTTGCGGTATACCCTCAAGTAGCACTAGTAGTAGTTGGCGCTTGTTACTTAGCGGCAGTGTGTGCTTGCGTGTGGAGTATAGTTCACTGGTAA                                                                                                                                                                                                                                                                                                                                 >  1:316360/1‑301`  
     `gacaGGAGGAAACACCTGATGGCTCTTTGCGGCACCCATCGTTCGTAATGTTCCGTGGCACCGAGGACAACCCTCAAGAGAAAATGTAATCACACTGGCTCACCTTCGGGTGGGCCTTTCTGCGTTTATAAGGAGACACTTTATGTTTAAGAAGGTTGGTAAATTCCTTGCGGCTTTGGCAGCTATCCTGACGCTTGCGTATATTCTTGCGGTATACCCTCAAGTAGCACTAGTAGTAGTTGGCGCTTGTCACTTAGCGGCAGTGTGTGCTTGCGTGTGGAGTATAGGTAACTGGTAATAC                                                                                                                                                                                                                                                                                                                              <  1:181685/297‑1`  
     `ACATGGAGGAAACACCTGATGGCTCTTTGCGGCACCCATCGTTCGTAATGTTCCGTGGCACCGAGGACAACCCTCAAGAGAAAATGTAATCACACTGGCTCACCTTCGGGTGGGCCTTTCTGCGTTTATAAGGAGACACTTTATGTTTAAGAAGGTTGGTAAATTCCTTGCGGCTTTGGCAGCTATCCTGACGCTTGCGTATATTCTTGCGGTATACCCTCAAGTAGCACTAGTAGTAGTTGTCGCTTGTTACTTAGCGGCAGTGTGTGCTTGCGTGTGGAGTATCGTTAACTGGTAATAC                                                                                                                                                                                                                                                                                                                              >  1:74514/1‑301`  
     `ACATGGAGGAAACACCTGATGGCTCTTTGCGGCACCCATCGTTCGTAATGTTCCGTGGCACCGAGGACAACCCTCAAGAGAAAATGTAATCACACTGGCTCACCTTCGGGTGGGCCTTTCTGCGTTTATAAGGAGACACTTTATGTTTAAGAAGGTTGGTAAATTCCTTGCGGCTTTGGCAGCTATCCTGACGCTTGCGTATATTCTTGCGGTATACCCTCAAGTAGCACTAGTAGTAGTTGGCGCTTGTTACTTAGCGGCAGTGTGTGCTTGCGTGTGGAGTATAGTTAACTGGTAATAC                                                                                                                                                                                                                                                                                                                              >  1:28890/1‑301`  
     `ACATGGAGGAAACACCTGATGGCTCTTTGCGACACCCATCGTTCGTAATGTTCCGTGGCACCGAGGACAACCCTCAAGAGAAAATGTAATCACACTGGCTCACCTTCGGGTGGGCCTTTCTGCGTTTATAAGGAGACACTTTATGTTTAAGAAGGTTGGTAAATTCCTTGCGGCTTTGGCAGCTATCCTGACGCTTGCGTATATTCTTGCGGTATACCCTCAAGTAGCACTAGTAGTAGTTGGCGCTTGTTACTTAGCGGCAGTGTGTGCTTGCGTGTGGAGTATAGTTAACTGGTAATAC                                                                                                                                                                                                                                                                                                                              <  1:119555/301‑1`  
      `acaGGAGGAAACACCTGATGGCTCTTTGCGGCACCCATCGTTCGTAATGTTCCGTGGCACCGAGGACAACCCTCAAGAGAAAATGTAATCACACTGGCTCACCTTCGGGTGGGCCTTTCTGCGTTTATAAGGAGACACTTTATGTTTAAGAAGGTTGGTAAATTCCTTGCGGCTTTGGCAGCTATCCTGACGCTTGCGCATATTCTTGCGGTATACCCTCAAGTAGCACTAGTAGTAGTTGGCGCTTGTTACTTAGCGGCAGTGTGTGCTTGCGTGTGGAGTATAGTTAACTGGTAATACG                                                                                                                                                                                                                                                                                                                             <  1:291888/298‑1`  
      `CATGGAGGAAACACCTGATGGCTCTTTGCGGCACCCATCGTTCGTAATGTTCCGTGGCACCGAGGACAACCCTCAAGAGAAAATGTAATCACACTGGCTCACCTTCGGGTGGGCCTTTCTGCGTTTATAAGGAGACACTTTATGTTTAAGAAGGTTGGTAAATTCCTTGCGGCTTTGGCAGCTATCCTGACGCTTGCGTATATTCTTGCGGTATACCCTCAAGTAGCACTAGTAGTAGTTGGCGCTTGTTACTTAGCGGCAGTGTGTGCTTGCGTGTGTAGTATAGTTAACTGGTAATACG                                                                                                                                                                                                                                                                                                                             <  1:337591/301‑1`  
       `tcggagatgggaataagagacagTTTTCGGCACCCATCGTTCGTAATGTTCCGTGGCACCGAGGACAACCCTCAAGAGAAAATGTAATCACACTGGCTCACCTTCGGGTGGGCCTTTCTGCGTTTATAAGGAGACACTTTATGTTTAAGAAGGTTGGTAAATTCCTTGCGGCTTTGGCAGCTATCCTGACGCTTGCGTATATTCTTTCGGTATACCCTCAAGTAGCACTAGTAGTAGTTGGCGCTTGTTACTTAGCGGCAGTGTGTGCTTGCGTGTGGAGTATAGTTAACTGGTAATACGA                                                                                                                                                                                                                                                                                                                            <  1:301438/278‑1`  
       `ATGTAGGAAACACCTGATGGCTCTTTGCGGCACCCATCGTTCGTAATGTTCCGTGGCACCGAGGACAACCCTCAAGAGAAAATGTAATCACACTGGCTCACCTTCGGGTGGGCCTTTCTGCGTTTATAAGGAGACACTTTATGTTTAAGAAGGTTGGTAAATTCCTTGCGGCTTTGGCAGCTATCCTGACCCTTGCGTATATTCTTGCGGTATACCCTCAAGTAGCACTAGTAGTAGTTGGCGCTTGTTACTTACCGGCCGTGTGTGCTTGCGTGTGGTGTATAGTTAACTGGTAATACtg                                                                                                                                                                                                                                                                                                                            >  1:123450/1‑299`  
        `aGGAGGAAACACCTGATGGCTCTTTGCGGCACCCATCGTTCGTAATGTTCCGTGGCACCGAGGACAACCCTCAAGAGAAAATGTAATCACACTGGCTCACCTTCGGGTGGGCCTTTCTGCGTTTATAAGGAGACACTTTATGTTTAAGAAGGTTGGTAAATTCCTTGCGGCTTTGGCAGCTATCCTGACGCTTGCGTATATTCTTGCGGTATACCCTCAAGTAGCACTAGTAGTAGTTGGCGCTTGTTACTTAGCGGCAGTGTGTGCTTGCGTGTGGAGTATAGTTAACTGGTAATACGAC                                                                                                                                                                                                                                                                                                                           <  1:227979/300‑1`  
          `GAGGAAACACCTGATGGCTCTTTGCGGCACCCATCGTTCGTAATGTTCCGTGGCACCGAGGACAACCCTCAAGAGAAAATGTAATCACACTGGCTCACCTTCGTGTGGGCCTTTCTGCGTTTATAAGGCGACACTTTATGTTTAAGAAGGTTGGTAAATTCCTTGCGGCTTTTGCCGCTATCCTGACGCTTGCGTATATTCTTGCGGTATACCCTCAAGTAGCACTAGTAGTAGTTGGCGCTTGTTACTTAGCGGCAGTGTGTGCTTGCGTGTGGAGTCTAGTTAACTGGTAATACGACTC                                                                                                                                                                                                                                                                                                                         >  1:102072/1‑301`  
          `GAGGAAACACCTGATGGCTCTTTGCGGCACCCATCGTTCGTAATGTTCCGTGGCACCGAGGACAACCCTCAAGAGAAAATGTAATCACACTGGCTCACCTTCGGGTGGGCCTTTCTGCGTTTATAAGGAGACACTTTATGTTTAAGAAGGTTGGTAAATTCCTTGCGGCTTTGGCAGCTATCCTGACGCTTGCGTATATTCTTGCAGTATACCCTCAAGTAGCACTAGTAGTAGTTGGCGCTTGTTACTTAGCGGCAGTGTGTGCTTGCGTGTGGAGTATAGTTAACTGGTAATACGACTC                                                                                                                                                                                                                                                                                                                         >  1:159883/1‑301`  
            `GGAAACACCTGATGGCTCTTTGCGGCACCCATCGTTCGTAATGTTCCGTGGCACCGAGGACAACCCTCAAGAGAAAATGTAATCACACTGGCTCACCTTCGGGTGGGCCTTTCTGCGTTTATAAGGAGACACTTTATGTTTAAGAAGGTTGGTAAATTCCTTGCTGCTTTTGCAGCTATCCTGACGCTTGCGTATATTATTGCGTTATACCCTCAAGTATCACTAGTAGTAGTTGGAGCTTGTTACTTAGCTGCAGTGTGTGCTTGCGTGTGGAGTTTAGTTAACTGGTAATACGACTCAC                                                                                                                                                                                                                                                                                                                       >  1:118024/1‑301`  
            `GGAAACACCTGATGGCTCTTTGCGGCACCCATCGTTCGTAATGTTCCGTGGCACCGAGGACAACCCTCAAGAGAAAATGTAATCACACTGGCTCACCTTCGGGTGGGCCTTTCTGCGTTTATAAGGAGACACTTTATGTTTAAGAAGGTTGGTAAATTCCTTGCGGCTTTGGCAGCTATCCTGACGCTTGCGTATATTCTTGCGGTATACCCTCAAGTAGCACTAGGAGTAGTTGGCGCTTGTTACTTAGCGGCAGTGTGTGCTTGCGTGTGGAGTATAGTTAACTGGTAATACGACTCAC                                                                                                                                                                                                                                                                                                                       >  1:170961/1‑301`  
              `AAACACCTGATGGCTCTTTGCGGCACCCATCGTTAGTAATGTTCCGTGGCACCGAGGACAACCCTCAAGAGAAAATGTAATCACACTGGCTCACCTTCGGGTGGGCCTTTCTGCGTTTATAAGGAGACACTTTATGTTTAAGAAGGTTGGTAAATTCCTTGCGGCTTTGGCAGCTATCCTGACGCTTGCATATATTCTTGCGGTATACCCTCAAGTAGCACTAGTAGTAGTTGGCGCTTGTTACTTAGCGGCAGTGTGTGCTTGCGTGTGGAGTATAGTTAACTGGTAATACGACTCACTA                                                                                                                                                                                                                                                                                                                     >  1:88878/1‑301`  
                 `CACCTGATGGCTCTTTGCGGCACCCATCGTTCGTAATGTTCCGTGGCACCGAGGACAACCCTCAAGAGAAAATGTAATCACACTGGCTCACCTTCGGGTGGGCCTTTCTGCGTTTATAAGGAGACACTTTATGTTTAAGAAGGTTGGTAAATTCCTTGCGGCTTTGGCAGCTATCCTGACGCTTGCGTATATTCTTGCGGTATACCCTCAAGTAGCACTAGTAGTAGTTGGCGCTTGTTACTTAGCGGCAGTGTGTGCTTGCGTGTGGAGTATAGTTAACTGGTAATACGACctgtctctt                                                                                                                                                                                                                                                                                                                  >  1:100153/1‑292`  
                 `CACCTGATGGCTCTTTGCGGCACCCATCGTTCGTAATGTTCCGAGGCACCGAGGACAACCCTCAAGAGAAAATGTAATCACACTGGCTCACCTTCGGGCGGACCTTTCTGCGTTTATAAGGAGACACTTTAGGTTTAAGAAGGTTGGTAAATTCCTTGCGGCTTTGGCAGCTATCCTGACGCCTGAGTATATCATAGCGGTATACCCTCAAGTAGCACTAGTAGTAGTTGGCGCTTGTTACTTAGCGGCAGTGTGCGCTTGCGAGTGGAGTATAGTTAACTGGTAATATGACTTACTAAAG                                                                                                                                                                                                                                                                                                                  >  1:56438/1‑301`  
                  `cCCTGATGGCTCTTTGTGGCACCCATCGTTCGTAATGTTCCGTGGCACCGAGGACAACCCTCAAGAGAAAATGTAATCACACTGGCTCACCTTCGGGTGGGCCTTTCTGCGTTTATAAGGAGACACTTTATGTTTAAGAAGGTTGGTAAATTCCTTGCGGCTTTGGCAGCTATCCTGACGCTTGCGTATATTCTTGCGGTATACCCTCAAGTAGCACTAGTAGTAGTTGGCGCTTGTTACTTAGCGGCAGTGTGTGCTTGCGTGTGGAGTATAGTTAACTGGTAATACGACTCACTAAAGG                                                                                                                                                                                                                                                                                                                 <  1:106523/300‑1`  
                  `cCCTGATGGCTCTTTGCGGCACCCATCGTTCGTAATGTTCCGTGGCACCGAGGACAACCCTCAAGAGAAAATGTAATCCCACTGGCTCACCTTCGGGTGGGCCTTTCTGCGTTTATAAGGAGACTCTTTATGTTTAAGAAGGTTGGTAAATTCCTTGCGGCTTTGGCAGCTATCCTGACGCTTGCGTATATTCTTGCGGTATACCCTCAAGTAGCACTAGTAGTAGTTGGCGCTTGTTACTTAGCGGCAGTGTGTGCTTGCGTGTGGAGTATAGTTAACTGGTAATACGACTCACTAAAGG                                                                                                                                                                                                                                                                                                                 <  1:91348/300‑1`  
                  `cCCTGATGGCTCTTTGCGGCACCCATCGTTCGTAATGTTCCGTGGCACCGAGGACAACCCTCAAGAGAAAATGTAATCACACTGGCTCACCTTCGGGTGGGCCTTTCTGCGTTTATAAGGAGACACTTTATGTTTAAGAAGGTTGGTAAATTCCTTGCGGCTTTGGCAGCTATCCTGACGCTTGCGTATATTCTTGCGGTATACCCTCAAGTAGCACTAGTAGTAGTTGGCGCTTGTTACTTAGCGGCAGTGTGTGCTTGCGTGTGGAGTATAGTTAACTGGTAATACGACTCACTAAAGG                                                                                                                                                                                                                                                                                                                 <  1:20139/300‑1`  
                  `cCCTGATGGCTCTTTGCGGCACCCATCGTTCGTAATGTTCCGTGGCACCGAGGACAACCCTCAAGAGAAAATGTAATCACACTGGCTCACCTTCGGGTGGGCCTTTCTGCGTTTATAAGGAGACACTTTATGTTTAAGAAGGTTGGTAAATTCCTTGCGGCTTTGGCAGCTATCCTGACGCTTGCGTATATTCTTGCGGTATACCCTCAAGTAGCACTAGTAGTAGTTGGCGCTTGTTACTTAGCGGCAGTGTGTGCTTGCGTGTGGAGTATAGTTAACTGGTAATACGACTCACTAAAGG                                                                                                                                                                                                                                                                                                                 <  1:197713/300‑1`  
                  `cCCTGATGGCTCTTTGCGGCACCCATCGTTCGTAATGTTCCGTGGCACCGAGGACAACCCTCAAGAGAAAATGTAATCACACTGGCTCACCTTCGGGTGAGCCTTTCTGCGTTTATAAGGAGACACTTTATGTTTAAGAAGGTTGGTAAATTCCTTGCGGCTTTGGCAGCTATCCTGACGCTTGCGTATATTCTTGCGGTATACCCTCAAGTAGCACTAGTAGTAGTTGGCGCTTGTTACTTAGCGGCAGTGTGTGCTTGCGTGTGGAGTATAGTTAACTGGTAATACGACTCACTAAAGG                                                                                                                                                                                                                                                                                                                 <  1:321120/300‑1`  
                  `cCCTGATGGCGCTGTGCGGCACCCATCGTTCGTAATGTTCCGTGGCACCGAGGACAACCCTCAAGAGAAAATGTAATCACACTGGCTCACCTTCGGGTGGGCCTTTCTGCGTTTATAAGGATACACTTTATGTTTAAGAAGGTTGGTAAATTCCTTGCGGCTTTGGCAGCTATCCTGACGCTTGCGTATATTCTTGCGGTATACACTCAAGTAGCACTAGTAGTAGTTGGCGCTTGTTTCTTAGCGGCAGTGTGTGCTTGCGTGTGGAGTATAGTTAACTGGTAATACGACTCACTAAAGG                                                                                                                                                                                                                                                                                                                 <  1:63507/300‑1`  
                  `ACCTGATGGCTCTTTGCGGCACCCATCGTTCGTAATGTTCCGTGGCACCGAGGACAACCCTCAAGAGAAAATGTAATCACACTGGCTCACCTTCGGGTGGGCCTTTCTGCGTTTATAAGGAGACACTTTATGTTTAAGAAGGTTGGTAAATTCCTTGCGGCTTTGGCAGCTATCCTGACGCTTGCGTATATTCTTGCGGTATACCCTCAAGTAGCACTAGTAGTAGTTGGCGCTTGTTACTTAGCGGCAGTGTGTGCTTGCGTGTGGAGTATAGTTAACTGGTAATACGACTCACTAAAGG                                                                                                                                                                                                                                                                                                                 <  1:58745/301‑1`  
                  `ACCTGATGGCTCTTTGCGGCACCCATCGTTCGTAATGTTCCGTGGCACCGAGGACAACCCTCAAGAGAAAATGTAATCACACTGGCTCACCTTCGGGTGGGCCTTTCTGCGTTTATAAGGAGACACTTTATGTTTAAGAAGGTTGGTAAATTCCTTGCGGCTTTGGCAGCTATCCTGACGCTTGCGTATATTCTTGCGGTATACCCTCAAGTAGCACTAGTAGTAGTTGGCGCTTGTTACTTAGCGGCAGTGTGTGCTTGCGTGTGGAGTATAGTTAACTGGTAATACGACTCACTAAAGG                                                                                                                                                                                                                                                                                                                 <  1:248767/301‑1`  
                  `ACCTGATGGCTCTTTGCGGCACCCATCGTTCGTAATGTTCCGTGGCACCGAGGACAACCCTCAAGAGAAAATGTAATCACACTGGCTCACCTTCGGGTGGGCCTTTCTGCGTTTATAAGGAGACACTTTATGTTTAAGAAGGTTGGTAAATTCCTTGCGGCTTTGGCAGCTATCCTGACGCTTGCGTATATTCTTGCGGTATACCCTCAAGTAGCACTAGTAGTAGTTGGCGCTTGTTACTTAGCGGCAGTGTGTGCTTGCGTGTGGAGTATAGTTAACTGGTAATACGACTCACTAAAGG                                                                                                                                                                                                                                                                                                                 <  1:131423/301‑1`  
                  `ACCTGATGGCTCTTTGCGGCACCCATCGTTCGTAATGTTACGTGGCACCGAGGACAACCCTCAAGAGAAAATGTAATCACACTGGCTCACCTTCGGGTGGGCCTTTCTGCGTTTATAAGGAGACACTTTATGTTTAAGAAGGTTGGTAAATTCCTTGCGGCTTTGGCAGCTATCCTGACGCTTGCGTATATTCTTGCGGTATACCCTCAAGTAGCACTAGTAGTAGTTGGCGCTTGTTACTTAGCGGCAGTGTGTGCTTGCGTGTGGAGTATAGTTAACTGGTAATACGACTCACTAAAGG                                                                                                                                                                                                                                                                                                                 <  1:76424/301‑1`  
                  `ACCTGATGGCTCTTTGCGGCACCCATCATTCGTAATGTTCCGTGGCACCGAGGACAACCCTCAAGAGAAAATGTAATCACACTGGCTCACCTTCGGGTGGGCCTTTCTGCGTTTATAAGGAGACGCTTTATGTTTAAGAAGGTTGGTAAATTCCGTGCGGCTTTGGCAGCTATCCTGACGCGTGCGTATATTCTTGCGGTATACCCTCAAGTAGCACTAGTAGTAGTTGGCGCTTGTTACTTAGCGGCAGTGTGTGCTTGCGTGTGGAGTATAGTTAACTGGTAATACGACTCACTAAAGG                                                                                                                                                                                                                                                                                                                 <  1:264587/301‑1`  
                   `CCTGATGGCTCTTTGCGGCACCCATCGTTCGTAATGTTCCGTGGCACCGAGGACAACCCTCAAGAGAAAATGTAATCCCACTGGCTCACCTTCGGGTTGGCCTTTCTGCGTTTATAATGAGACACTTTATGTTTAAGAAGGTTGGTAAATTCCTTGCGGCTTTGGCATCTATCCTGACCCTTGCGTATATTCTTTCGTTATACCCTCAAGTAGCCCTTGTAGTAGTTTGCGCTTGTTACTTATCGGCAGTGTGTGCTTGCGTGTGGAGTCTAGTTAACTGGTAATACGACTCACTAAAGGA                                                                                                                                                                                                                                                                                                                >  1:19538/1‑301`  
                   `CCTGATGGCTCTTTGCGGCACCCATCGTTCGTAATGTTCCGTGGCACCGAGGACAACCCTCAAGAGAAAATGTAATCACACTGGCTCACCTTCGGGTGGGCCTTTCTGCGTTTATAAGGAGACACTTTATGTTTAAGAAGGTTGGTAAATTCCTTGCGGCTTTGGCAGCTATCCTGACGCTTGCGTATATTCTTGCGGTATACCCTCAAGTAGCACTAGTAGTAGTTGGCGCTTGTTACTTAGCGGCAGTGTGTGCTTGCGTGTGGAGTATAGTTAACTGGTAATACGACTCACTAAAGGA                                                                                                                                                                                                                                                                                                                >  1:273010/1‑301`  
                   `CCTGATGGCTCTTTGCGGCACCCATCGTTCGTAATGTTCCGTGGCACCGAGGACAACCCTCAAGAGAAAATGTAATCACACTGGCTCACCTTCGGGTGGGCCTTTCTGCGTTTATAAGGAGACACTTTATGTTTAAGAAGGTTGGTAAATTCCTTGCGGCTTTGGCAGCTATACTGACGCTTGCGTATATTCTTGCGGTATACCCTAAAGTAGCACTAGTAGTAGTTGGCGCTTGTTACTTAGCGGCAGTGTGTGCTTGCGTGTGGAGTATAGTTAACTGGTAATACGACTCACTAAAGGc                                                                                                                                                                                                                                                                                                                >  1:243826/1‑300`  
                   `CCTGATGGCTATTTGCGGCACCCATCGTTCGTAATGTTCCGTGGAACCGAGGACAACCCTCAAGAGAAAATGTAATCACACTGGCTCACCTTCGGGTGGGCCTTTCTGCGTTTATAAGGAGACACTTTATGTTTAAGAAGGTTGGTAAATTCCTTGCGGCTTTGGCAGCTATCCTGACGCTTGCGTATATTCTTGCGGTATACCCTCAAGTAGCACTAGTAGTAGTTGGCGCTTGTTACTTAGCGGCAGTGTGTGCTTGCGTGTGGAGTATAGTTAACTGGTAATACGACTCACTAAAGGA                                                                                                                                                                                                                                                                                                                >  1:305437/1‑301`  
                     `TGATGGCTCTTTGCGGCACCCATCGTTCATAATGTTCCGTGGCACCGAGGACAACCCTCAAGAGAAAATGTAATCACACTGGCTCACCTTCGGGTGGGCCTTTCTGCGTTTATAAGGAGACACTTTATGTTTAAGAAGGTTGGTAAATTCCTTGCGGCTTTGGCAGCTATCCTGACTCTTGCGTATATTCTTGCGGTATACCCTCAAGTAGCACTAGTAGTAGTTGGCGCTTGTTACTTAGCGGCAGTGTGTGCTTGCGTGTGGAGTATAGTTAACTGGTAATACGACTCACTAAAGGctg                                                                                                                                                                                                                                                                                                              >  1:239876/1‑298`  
                          `GCTCTTTGCGGCACCCATCGTTCGTAATGTTCCGTGGCACCGAGGACAACCCTCAAGAGAAAATGTAATCACACTGGCTCACCTTCGGGTGGGCCTTTCTGCGTTTATAAGGAGACACTTTATGTTTAAGAAGGTTGGTAAATTCCTTGCGGCTTTGGCAGCTATCCTGACGCTTGCGTATATTCTTGCGGTATACCCTCAAGTAGCACTAGTAGTAGTTGGCGCTTGTTACTTAGCGGCAGTGTGTGCTTGCGTGTGGAGTATAGTTAACTGGTAATACGACTCACTAAAGGctgtctct                                                                                                                                                                                                                                                                                                         >  1:284258/1‑293`  
                             `CTTTGCGGCACCCATCGTTCGTAATGTTCCGTGGCACCGAGGACAACCCTCAAGAGAAAATGTAATCACACTGGCTCACCTTCGGGTGGGCCTTTCTGCGTTTATAAGGAGACACTTTATGTTTAAGAAGGTTGGTAAATTCCTTGCGGCTTTGGCAGCTATCCTGACGCTTGCGTATATTCTTGCGGTATACCCTCAAGTAGCACTAGTAGTAGTTGGCGCTTGTTACTTAGCGGCAGTGTGTGCTTGCGTGTGGAGTATAGTTAACTGGTAATACGACTCACTAAAGGctgtctcttat                                                                                                                                                                                                                                                                                                      >  1:222915/1‑290`  
                              `TTTGAGGCACCCATCGTTCGTAATGTTCAGTGGCACCGAGGACAACCCTCAAGAGAAAATGTAATCACACTGGCTCACCTTCGGGTGGGCCTTTCTGCGTTTATAAGGAGACACTTTATGTTTAAGAAGGTTGGTAAATTCCTTGCGGCTTTGGCAGCTATCCTGACGCTTGCGTATATTCTTGCGGTATACCCTCAAGTAGCACTAGTAGTAGTTGGCGCTTGTTACTTAGCGGCAGTGTGTGCTTGCGTGTGGAGTATAGTTAACTGGTAATACGACTCACTAAAGGAGACACTATctt                                                                                                                                                                                                                                                                                                     <  1:121334/301‑4`  
                                    `GCACCCATCGTTCGTAATGTTCCGTGGCACCGAGGACAACCCTCAAGAGAAAATGTAATCACACTGGCTCACCTTCGGGTGGGCCTTTCTGCGTTTATAAGGAGACACTTTATGTTTAAGAAGGTTGGTAAATTCCTTGCGGCTTTGGCAGCTATCCTGACGCTTGCGTATATTCTTGCGGTATACCCTCAAGTAGCACTAGTAGTAGTTGGCGCTTGTTACTTAGCGGCAGTGTGTGCTTGCGTGTGGAGTATAGTTAACTGGTAATACGACTCACTAAAGGctgtctcttatacacatc                                                                                                                                                                                                                                                                                               >  1:96288/1‑283`  
                                                                                            `aacagCACTGACTCACCTTCTGTTGGGCCTTTCTGCGTTTATAAGGTGCCCCTTTATGTTTAAGAAGGTTGGTAAATTCCTTGCGGCTTTGGCAGCTATTCTGTCGCTTGCGTATATTCTTGCGGTATACCCTCGAGTATCACTAGTAGTAGTTGGCGCTTGTTACTTCGCGGCAGTGTGTGCTTGCGTGTGGAGTATAGTTAACTGTTAATACGCCTCACTAAATGATACACTATATGTTTCTACTTCATTACAACAAAAGCGTTAAGAATTTCACGGTTCGCCGTGCTGACCATTCAAT                                                                                                                                                                                                                                       <  1:110522/296‑1`  
                                                                                              `TCACACTGGCTCACCTTCGGGTGGGCCTTTCTGCGTTTATAAGGAGACACTTTATGTTTAAGAAGGTTGGTAAATTCCTTGCGGCTTTGGCAGCTATCCTGACGCTTGCGTATATTCTTGCGGTATACCCTCAAGTAGCACTAGTAGTAGTTGGCGCTTGTTACTTAGCGGCAGTGTGTGCTTGCGTGTGGAGTATAGTTAACTGGTAATACGACTAACTAAAGGAGACACTATATGTTTCGACTTCATTACAACAAAAGCGTTAAGAATTTCACGGTTCGCCGTGCTGACCGTTCAATCG                                                                                                                                                                                                                                     <  1:269745/301‑1`  
                                                                                                           `CCTTCGGGTGGGCCTTTCTGCGTTTATAAGGAGACACTTTATGTTTAAGAAGGTTGGTAAATTCCTTGCGGCTTTGGCAGCTATCCTGACGCTTGCGTATATTCTTGCGGTATACCCTCAAGTAGCACTAGTAGTAGTTGGCGCTTGTTACTTAGCGGCAGTATGTGCTTGCGTGTGGAGTATAGTTAACTGGTAATACGACTCACTAAAGGAGACACTATATGTTTCGACTTCATTACAACAAAAGCGTTAAGAATTTCACGGTTCGCCGTGCTGACCGTTCAATCGTATGTGCGAGCGA                                                                                                                                                                                                                        >  1:80736/1‑301`  
                                                                                                                                                                                             `ATCCTGACGCTTGCGTATATTCTTGCGGTATACCCTCAAGTAGCACTAGTAGTAGTTGGCGCTTGTTACTTAGCGGCAGTGTGTGCTTGCGTGTGGAGTATAGTTAACTGGTAATACGACTCACTAAAGGAGACACTATATGTTTCGACTTCATTACAACAAAAGCGTTAAGAATTTCACGGTTCGCCGTGCTGACCGTTCAATCGTATGTGCGAGCGAGCGCCGAGCTAAGATACCTCTTACTGGTAACACAGTTCCTTTGGCACCGAGCGTCCACATCATTATCACCCGTGGTGACTTT                                                                                                                                      >  1:146278/1‑301`  
                                                                                                                                                                                                             `ATATTCTTGCGGTATACCCTCAAGTAGCACTAGTAGTAGTTGGCGCTTGTTACTTAGCGGCAGTGTGTGCTTGCGTGTGGAGTATAGTTAACTGGTAATACGACTCACTAAAGGAGACACTATATGTTTCGACTTCATTACAACAAAAGCGTTAAGAATTTCACGGTTCGCCGTGCTGACCGTTCAATCGTATGTGCGAGCGAGCGCCGAGCTAAGATACCTCTTATTGGTAACACAGTTCCTTTGGCACCGAGCGTCAACATCATTATCACCCGTGGTGACTTTGAGAAAGCctgtctct                                                                                                                      >  1:139687/1‑293`  
                                                                                                                                                                                                                     `GCGGTATACCATCAAGTAGCACTAGTAGTAGTTGGCGCTTGTTACTTAGCGGCAGTGTGTGCTTGCGTGTGGAGTATAGTTAACTGGTAATACGACTCACTAAAGGAGACACTATATGTTTCGACTTCATTACAACAAAAGCGTTAAGAATTTCACGGTTCGCCGTGCTGACCGTTCAATCGTATGTGCGAGCGAGCGCCGAGCTAAGATACCTCTTATTGGTAACACAGTTCCTTTGGCACCGAGCGTCCACATCATTATCACCCGTGGTGACTTTGAGAAAGCAATAGACAAG‑AAACGT                                                                                                             >  1:293696/1‑301`  
                                                                                                                                                                                                                       `GGTATACCCTCAAGTAGCACTAGTAGTAGTTGGCGCTTGTTACTTAGCGGCAGTGTGTGCTTGCGTGTGGAGTATAGTTAACTGGTAATACGACTCACTAAAGGAGACACTATATGTTTCGACTTCATTACAACAAAGGCGTTAAGAATTTCACGGTTCGCCGTGCTGACCGTTCAATCGTATGTGCGAGCGAGCGCCGAGCTAAGATACCTCTTATTGGTAACACAGTTCCTTTGGCACCGAGCGTCCACATCATTATCACCCGTGGTGACTTTGAGAAAGCAATAGACAAG‑AAACGTCC                                                                                                           >  1:136912/1‑301`  
                                                                                                                                                                                                                                             `GTAGTAGTTGGCGCTTGTTACTTAGCGGCAGTGTGTGCTTGCGTGTGGAGTATAGTTAACTGGTAATACGACTCACTAAAGGAGACACTATATGTTTCGACTTCATTACAACAAAAGCGTTAAGAATTTCACGGTTCGCCGTGCTGACCGTTCAATCGTATGTGCGAGCGAGCGCCGAGCTAAGATACCTCTTATTGGTAACACAGTTCCTTTGGCACCGAGCGTCCACATCATTATCACCCGTGGTGACTTTGAGAAAGCAATAGACAAG‑AAACGTCCGGTTCTTAGTGTGGCctgtctc                                                                                     >  1:187532/1‑294`  
                                                                                                                                                                                                                                             `GTAGTAGTTGGCGCTTGTTACTTAGCGGCAGTGTGTGCTTGCGTGTGGAGTATAGTTAACTGGTAATACGACTCACTAAAGGAGACACTATATGTTTCGACTTCATTACAACAAAAGCGTTAAGAATTTCACGGTTCGCCGTGCTGACCGTTCAATCGTATGTGCGAGCGAGCGCCGAGCTAAGATACCTCTTATTGGTAACACAGTTCCTTTGGCACCGAGCGTCCACATCATTATCACCCGTGGTGACTTTGAGAAAGCAATAGACAAG‑AAACGTCCGGTTCTTAGTGTGGCAGTGACC                                                                                     <  1:274414/301‑1`  
                                                                                                                                                                                                                                                                                `gGCTTGCGTGTGGAGTATAGTTAACTGGTAATACGACTCACTAAAGGAGACACTATATGTTTCGACTTCATTACAACAAAAGCGTTAAGAATTTCACGGTTCGCCGTGCTGACCGTTCAATCGTATGTGCGAGCGAGCGCCGAGCTAAGATACCTCTTATTGGTAACACAGTTCCTTTGGCACCGAGCGTCCACATCATTATCACCCGTGGTGACTTTGAGAAAGCAATAGACAAG‑AAACGTCCGGTTCTTAGTGTGGCAGTGACCCGCTTCCCGTTCGTCAGTCTGTTACTCAAACAAAT                                                  <  1:334660/300‑1`  
                                                                                                                                                                                                                                                                                      `agagacagGTATATTTAACTGGTAATACGACTCACTAAAGGAGACACTATATGTTTCGACTTCATTACAACAAAAGCGTTAAGAATTTCACGGTTCGCCGTGCTGACCGTTCAATCGTATGTGCGAGCGAGCGCCGAGCTAAGATACCTCTTATTGGTAACACAGTTCCTTTGGCACCGAGCGTCCACATCATTATCACCCGTGGTGACTTTGAGAAAGCAATAGACAAG‑AAACGTCCGGTTCTTAGTGTGGCAGTGACCCGCTTCCCGTTCGTCCATCTGTTACTCAAACGAATCAAGGA                                            <  1:40177/293‑1`  
                                                                                                                                                                                                                                                                                                  `gggctcggagatgtgtataagagacagGGAGACACTATATGTTTCGACTTCATTACAACAAAAGCGTTAAGAATTTCACGGTTCGCCGTGCTGACCGTTCAATCGTATGTGCGAGCGAGCGCCGAGCTAAGATACCTCTTATTGGTAACACAGTTCCTTTGGCACCGAGCGTCCACATCATTATCACCCGTGGTGACTTTGAGAAAGCAATAGACAAG‑AAACGTCCGGTTCTTAGTGTGGCAGTGACCCGCTTCCCGTTCGTCCGTCTGTTACTCAAACGAATCAAGGAGGTGTTCTGATG                                <  1:184229/274‑1`  
                                                                                                                                                                                                                                                                                                  `gctcgggctcggagatgtgtataagagacAGACACTATATGTTTCGACTTCATTACAACAAAAGCGTTAAGAATTTCACGGTTCGCCGTGCTGACCGTTCAATCGTATGTGCGAGCGAGCGCCGAGCTAAGATACCTCTTATTGGTAACACAGTTCCTTTGGCACCGAGCGTCCACATCATTATCACCCGTGGTGACTTTGAGAAAGCAATAGACAAG‑AAACGTCCGGTTCTTAGTGTGGCAGTGACCCGCTTCCCGTTCGTCCGTCTGTTACTCAAACGAATCAAGGAGGTGTTCTGATG                                <  1:276994/272‑1`  
                                                                                                                                                                                                                                                                                                            `TAATACGACTCACTAAAGGAGACACTATATGTTTCGACTTCATTACAACAAAAGCGTTAAGAATTTCACGGTTCGCCGTGCTGACCGTTCAATCGTATGTGCGAGCGAGCGCCGAGCTAAGATACCTCTTATTGGTAACACAGTTCCTTTGGCACCGAGCGTCCACATCATTATCACCCGTGGTGACTTTGAGAAAGCAATAGACAAG‑AAACGTCCGGTTCTTAGTGTGGCAGTGACCCGCTTCCCGTTCGTCCGTCTGTTACTCAAACGAATCCAGGAGGTGTTCTGATGTGACTGTTAG                      >  1:280044/1‑301`  
                                                                                                                                                                                                                                                                                                            `TAATACGACTCACTAAAGGAGACACTATATGTTTCGACTTCATTACAACAAAAGCGTTAAGAATTTCACGGTTCGCCGTGCTGACCGTGAAATCGTATGTGCGAGCGAGCGCCGAGATAAGATACCTCTTAATGGTAACACAGATCCTTTGGCACCGAGCGTCCACATCATTATCACCCGTGGTGACTTTGAGAAAGCAATAGACAAG‑AAACGTCCGGTTATTAGTGTGGCAGTGACCCGCTTCCCGTTCGTCCGTCCGATACTCAAACGAATCAAGGAGGTGTTCTGATGGGACAGTTAG                      >  1:155087/1‑301`  
                                                                                                                                                                                                                                                                                                             `AATACGACTCACTAAAGGAGACACTATATGTTTCGACTTCATTACAACAAAAGCGTTAAGAATTTCACGGTTCGCCGTGCTGACCGTTCAATCGTATGTGCGAGCGAGCGCCGAGCTAAGATACCTCTTATTGGTAACACAGTTCCTTTGGCACCGAGCGTCCACATCATTATCACCCGTGGTGACTTTGAGAAAGCAATAGACAAG‑AAACGTCCGGTTCTTAGTGTGGCAGTGACCCGCTTCCCGTTCGTCCGTCTGTTACTCAAACGAATCAAGGAGGTGTTCTGATGGGACTGTTAGA                     <  1:340825/301‑1`  
                                                                                                                                                                                                                                                                                                              `taagagacagCTAAAGGAGACACTATATGTTTCGACTTCATTACAACAAAAGCGTTAAGAATTTCACGGTTCGCCGTGCTGACCGTTCAATCGTATGTGCGAGCGAGCGCCGAGCTAAGATACCTCTTATTGGTAACACAGTTCCTTTGGCACCGAGCGTCCACATCATTATCACCCGAGGTGACTTTGAGAAAGCAATAGACAAG‑AAACGTCCGGTTCTTAGTGTGGCAGTGACCCGCTTCCCGTTCGTCCGTCTGTTACTCAAACGAATCAAGGAGGTGTTCTGATGGGACTGTTAGAT                    <  1:163632/291‑1`  
                                                                                                                                                                                                                                                                                                              `gagacagTCACTAAAGGGGACACTATATGTTTCGACTTCATTACAACAAAAGCGTTAAGAATTTCACGGTTCGCCGTGCTGACCGTTCAATCGTATGTGCGAGCGAGCGCCGAGCTAAGATACCTCTTATTGGTAACACAGTTCCTTTGGCACCGAGCGTCCACATCATTATCACCCGTGGTGACTTTGAGAAAGCAATAGACAAG‑AAACGTCCGGTTCTTAGTGTGGCAGTGACCCGCTTCCCGTTCGTCCGTCTGTTACTCAAACGAATCAAGGAGGTGTTATGATGGGACTGTTAGAT                    <  1:233241/294‑1`  
                                                                                                                                                                                                                                                                                                              `agaaGACTCACTAAAGGGGAAACTATATGTTTCGACTTCATTACAACAAAAGCGTTAAGAATTTCACGGTTCGCCGTGCTGACCGTTCAATCGTATGTGCGAGCGAGCGCCGAGCTAATATACCTCTTATTGGTAACACAGTTCCTTTGGCACCGAGCGTACACATCATTATCACCCGTGGTGACTTTGAGAAAGCAATAGACAAG‑AAACGTCCGGTTCTTAGTGTGGCAGTGACCCGCTTCCCGTTCGTCCGTCTGTTACTCAAACGAATCAAGGAGGTGTTCTGATGGGACTGTTAGAT                    <  1:204175/297‑1`  
                                                                                                                                                                                                                                                                                                              `ATACTACTCACTAAAGGATACACTATATGTTTCGACTTCATTCCAACAAAAGCGTTAAGAATTGCACGGTTCGCCGGGCTGACCGTTAAATCGTATGTGCGAGCTAGCGCCGAGCTAAGATACCTCTTATTGGTAACACAGTTCCTTTGGCACCGAGCGTCCACATCATTATCACCCGTGGTGACTTTGAGAAAGCAATAGACAAG‑AAACGTCCGGTTCTTAGTGTGGCAGTGACCCGCTTCCCGTTCGTCCGTCTGTTACTCAAACGAATCAAGGAGGTGTTCTGATGGGACTGTTAGAT                    <  1:307746/301‑1`  
                                                                                                                                                                                                                                                                                                              `ATACGACTCACTAAAGGAGACACTATATGTTTCGACTTCATTACAACAAAAGCGTTAAGAATTTCACGGTTCGCCGTGCTGACCGTTCAATCGTATGTGCGAGCGAGCGCCGAGCTAAGATACCTCTTATTGGTAACACAGTTCCTTTGGCACCGAGCGTCCACATCATTATCACCCGTGGTGACTTTGAGAAAGCAATAGACAAG‑AAACGTCCGGTTCTTAGTGTGGCAGTGACCCGCTTCCCGTTCGTCCGTCTGTTACTCAAACGAATCAAGGAGGTGTTCTGATGGGACTGTTAGAT                    <  1:32710/301‑1`  
                                                                                                                                                                                                                                                                                                              `ATACGACTCACTAAAGGAGACACTATATGTTTCGACTTCATTACAACAAAAGCGTTAAGAATTTCACGGTTCGCCGTGCTGACCGTTCAATCGTATGTGCGAGCGAGCGCCGAGCTAAGATACCTCTTATTGGTAACACAGTTCCTTTGGCACCGAGCGTCCACATCATTATCACCCGTGGTGACTTTGAGAAAGCAATAGACAAG‑AAACGTCCGGTTCTTAGTGTGGCAGTGACCCGCTTCCCGTTCGTCCGTCTGTTACTCAAACGAATCAAGGAGGTGTTCTGATGGGACTGTTAGAT                    <  1:167888/301‑1`  
                                                                                                                                                                                                                                                                                                              `ATACGACTCACTAAAGGAGACACTATATGGTTCGACTTCATTACAACAAAAGCGTTAAGAATGTCACGGTTCGCCGTGCTGACCGTTCAATCGTATGTGCGAGCGAGCGCCGAGCTAAGATACCTCTTATTGGTAACACAGTTCCTTTGGCACCGAGCGTCCACATCATTATCACCCGTGGTGACTTTGAGAAAGCAATAGACAAG‑AAACGTCCGGTTCTTAGTGTGGCAGTGACCCGCTTCCCGTTCGTCCGTCTGTTACTCAAACGAATCAAGGAGGTGTTCTGATGGGACTGTTAGAT                    <  1:176366/301‑1`  
                                                                                                                                                                                                                                                                                                                `acgaggCACTAGAGGAGACACTCTATGTTTCGGCTTCATTACAACAAAAGCGGTAAGAATGTCACGGTTCGCCGTGCTGACCGTTCAAACGTATGTGCGAGCGAGCGCCGAGCTAAGATACCTCTTATTGGTAACACAGTTCCTTTGGCACCGAGCGTCCACATCATTATCACCCGTGGTGACTTTGAGAAAGCAATAGACAAG‑AAACGTCCGGTTCTTAGTGTGGCAGTGACCCGCTTCCCGTTCGTCCGTCTGTTACTCAAACGAATCAAGGAGGTGTTCTGATGGGACTGTTAGATGG                  <  1:51183/295‑1`  
                                                                                                                                                                                                                                                                                                                `ACGACTCACTAAAGGAGACACTATATGTTTCGACTTCATTACAACAAAAGCGTTAAGAATTTCACGGTTCGCCGTGCTGACCGTTCAATCGTATGTGCGAGCGAGCGCCGAGCTAAGATACCTCTTATTGGTAACACAGTTCCTTTGGCACCGAGCGTCCACATCATTATCACCCGTGGTGACTTTGAGAAAGCAATAGACAAG‑GAACGTCCTGTTCTTAGTGTGGCAGTGACCCGCTTCCCGTTCGTCCGTCTGTTACTCAAACGAATCAAGGAGGTGTTCTGATGGGACTGTTAGATGG                  <  1:194323/301‑1`  
                                                                                                                                                                                                                                                                                                                `ACGACTCACTAAAGGAGACACTATATGTTTCGACTTCATTACAACAAAAGCGTTAAGAATTTCACGGTTCGCCGTGCTGACCGTTCAATCGTATGTGCGAGCGAGCGCCGAGCTAAGATACCTCTTATTGGTAACACAGTTCCTTTGGCACCGAGCGTCCACATCATTATCACCCGTGGTGACTTTGAGAAAGCAATAGACAAG‑AAACGTCCGGTTCTTAGTGTGGCAGTGACCCGCTTCCCGTTCGTCCGTCTGTTACTCAAACGAATCAAGGAGGTGTTCTGATGGGACTGTTAGATGG                  <  1:297119/301‑1`  
                                                                                                                                                                                                                                                                                                                `ACGACTCACTAAAGGAGACACTATATGTTTCGACTTCATTACAACAAAAGCGTTAAGAATTTCACGGTTCGCCGTGCTGACCGTTCAATCGTATGTGCGAGCGAGCGCCGAGCTAAGATACCTCTTATTGGTAACACAGTTCCTTTGGCACCGAGCGTCCACATCATTATCACCCGTGGTGACTTTGAGAAAGCAATAGACAAG‑AAACGTCCGGTTCTTAGTGTGGCAGTGACCCGCTTCCCGTTCGTCCGTCTGTTACTCAAACGAATCAAGGAGGTGTTCTGATGGGACTGTTAGATGG                  <  1:7338/301‑1`  
                                                                                                                                                                                                                                                                                                                `ACGACTCACTAAAGGAGACACGATATGTGTCGACTTAATTACAACAAAAGCGTGAAGAATTTCACGGTTCGCCGTGCTGACCGTTCAATCGTATGTGCGAGCGAGCGCCGAGCTAAGATACCTCTGATTGGTAACACAGTTCCTTTGGCACCGAGCGTCAACAGCATTATCACCCGTGTTGACTTTGAGAAAGCAATAGACAAG‑AAACGTCCGGTTCTTAGTGTGGCAGTGACCCGCTTCCCGTTCGTCCGTCTGTTACTCAAACGAATCAAGGAGGTGTTCTGATGGGACTGTTAGATGG                  <  1:47270/301‑1`  
                                                                                                                                                                                                                                                                                                                 `tgtataagagacaggGACACTATATGTTTCGAATTCATTACAACAAAAGCGTTAAGAATTTCACGGTTCGCCGTGCTGACCGTTCAATCGTATGTGCGAGCGAGCGCCGAGCTAAGATACCTCTTATTGGTAACACAGTTCCTTTGGCACCGAGCGTCCACATCATTATCACCCGTGGTGACTTTGAGAAAGCAATAGACAAG‑AAACGTCCGGTTCTTAGTGTGGCAGTGACCCGCTTCCCGTTCGTCCGTCTGTTACTCAAACGAATCAAGGAGGTGTTCTGATGGGACTGTTAGATGGc                 <  1:192093/286‑2`  
                                                                                                                                                                                                                                                                                                                 `CGACTCACTAAAGGAGACACTATATGTTTCGACTTCATTACAACAAAAGCGTTAAGAATTTCACGGTTCGCCGTGCTGACCGTTCAATCGTATGTGCGAGCGAGCGCCGAGCTAAGATACCTCTTATTGGTAACACAGTTCCTTTGGCACCGAGCGTCCACATCATTATCACCCGTGGTGACTTTGAGAAAGCAATAGACAAG‑AAACGTCCGGTTCTTAGTGTGGCAGTGACCCGCTTCCCGTTCGTCCGTCGGTTACTCAAACGAATCAAGGAGGTGTTCTGATGGGACTGTTAGATGGg                 >  1:208432/1‑300`  
                                                                                                                                                                                                                                                                                                                  `GACTCACTAAAGGGGACACTATATGTTTCGACCTCATTACAACACAAGCGTTAAGAATTTCACGGTTCGCCGTGCTGACCGTTCAATCGTATGTGCGAGCGAGCGCCGAGCTAAGATACCTCTTATTGGTAACACAGTTCCTTTGGCACCGAGCGTCCACATCATTATCACCCGTGGTGACTTTGAGAAAGCAATATACAAG‑AAACGTCCGGTTCTTAGTGTGGAAGTGACCCGCTTCCCGTTCGTCCGTCTGTTACTCAAACGAATCAAGGAGGTGTTCTGATGGGACTGTTAGATGGTG                <  1:34677/301‑1`  
                                                                                                                                                                                                                                                                                                                    `CTCACTAAAGGAGACACTATATGTTTCGACTTCATTACAACAAAAGCGTTAAGAATTTCACGGTTCGCCGTGCTGACCGTTCAATCGTATGTGCGAGCGAGCGCCGAGCTAAGATACCTCTTATTGGTAACACAGTTCCTTTGGCACCGAGCGTCCACATCATTATCACCCGTGGTGACTTTGAGAAAGCAATAGACAAG‑AAACGTCCGGTTCTTAGTGTGGCAGTGACCCGCTTCCCGTTCGTCCGTATGTTACTCAAACGAATCAAGGAGGTGTTCTGATGGGACTGTTAGATGGTGAA              >  1:25723/1‑301`  
                                                                                                                                                                                                                                                                                                                      `CACTAAAGGAGACACTATATGTTTCGTCTTCATTACAACAAAAGCGTTAAGAATTTCACGGTTCGCCGTGCTGACCGTTCAATCGTATGTGCGAGCGAGCGCCGAGCTAAGATACCTCTTATTGGTAACACAGTTCCTTTGGCACCGAGCGTCCACATCATTATCACCCGTGGTGACTTTGAGAAAGCAATAGACAAG‑AAACGTCCGGTTCTTAGTGTGGCAGTGACCCGCTTCCCGTTCGTCCGTCTGTTACTCAAACGAATCAAGGAGGTGTTCTGATGctgtctcttatacacatctc            >  1:218764/1‑281`  
                                                                                                                                                                                                                                                                                                                      `CACTAAAGGAGACACTATATGTTTCGACTTCATTACAACAAAAGCGTTAAGAATTTCACGGTTCGCCGTGCTTACCGTTCAATCGTATGTGCGAGCGAGCGCCGAGCTAAGATACCTCTTATTGGTAACACAGTTCCTTTGGCACCGAGCGTCCACATCATTATCACCCGTGGTGACTTTGAGAAAGCAATATACAAG‑AAACGTCCGGTTCTTAGTGTGGCAGTGACCCGCTTCCCGTTCGTCCGTCTGTTACTCAAACGAATCAAGGAGGTGTTCTGActgtctcttatacacatctccg            >  1:247395/1‑279`  
                                                                                                                                                                                                                                                                                                                      `CACTAAAGGAGACACTATATGTTTCGACTTCATTACAACAAAAGCGTTAAGAATTTCACGGTTCGCCGTGCTTACCGTTCAATCGTATGTGCGAGCGAGCGCCGAGCTAAGATACCTCTTATTGGTAACACAGTTCCTTTGGCACCGAGCGTCCACATCATTATCACCCGTGGTGACTTTGAGAAAGCAATAGACAAG‑AAACGTCCGGTTCTTAGTGTGGCAGTGACCCGCTTCCCGTTCGTCCGTCTGTTACTCAAACGAATCAAGGAGGTGTACTGATGGGACTGTTAGATGGctgtct            >  1:71358/1‑295`  
                                                                                                                                                                                                                                                                                                                      `CACTAAAGGAGACACTATATGTTTCGACTTCATTACAACAAAAGCGTTAAGAATTTCACGGTTCGCCGTGCTGACCGTTCAATCGTATGTGCGAGCTAGCGCAGAGCTAAGATACCTCTTATTGGTAACACAGTTCCTTTGGCACCGAGCGTCCACATCATTATCACCCGTGGTGACTTTGAGAAAGCAATAGACAAG‑AAACGTCCGGTTCTTAGTGTGGCAGTGACACGCTTCCCGTTAGTCCGTCTGTTACCCAAACGAATCAAGGCGGTGTTCTGACGGGACTGTTAGATGGGGAAGC            >  1:109345/1‑301`  
                                                                                                                                                                                                                                                                                                                      `CACTAAAGGAGACACTATATGTTTCGACTTCATTACAACAAAAGCGTTAAGAATTTCACGGTTCGCCGTGCTGACCGTTCAATCGTATGTGCGAGCGAGCGCCGAGCTAAGATACCTCTTATTGGTAACACAGTTCCTTTGTCACCGAGCGTCCACATCATTATCACCCTTTGTTACTGTCAGAAAGCAATAGACACG‑AAACGTCCGGTTCTTAGTTTGGCATTACCCCGCTTCCCGCTCGTCCGTCTGTTCCTCAAACGCATCAAGGAGGTGTTCTGTTTTGACTGTTATATGGTcatgc            >  1:237296/1‑296`  
                                                                                                                                                                                                                                                                                                                      `CACTAAAGGAGACACTATATGTTTCGACTTCATTACAACAAAAGCGTTAAGAATTTCACGGTTCGCCGTGCTGACCGTTCAATCGTATGTGCGAGCGAGCGCCGAGCTAAGATACCTCTTATTGGTAACACAGTTCCTTTGGCACCGAGCGTCCACATCATTATCACCCGTGGTGACTTTGAGAAAGCAATAGACAAG‑AAACGTCCGGTTCTTAGTGTGGCAGTGACCCGCTTCCCGTTCGTCCGTCTGTTACTCAAACGAATCAAGGATGTGTTCTGATGGGACTGTTAGATGGTGAAGC            >  1:116357/1‑301`  
                                                                                                                                                                                                                                                                                                                      `CACTAAAGGAGACACTATATGTTTCGACTTCATTACAACAAAAGCGTTAAGAATTTCACGGTTCGCCGTGCTGACCGTTCAATCGTATGTGCGAGCGAGCGCCGAGCTAAGATACCTCTTATTGGTAACACAGTTCCTTTGGCACCGAGCGTCCACATCATTATCACCCGTGGTGACTTTGAGAAAGCAATAGACAAG‑AAACGTCCGGTTATTAGTGTGGCAGTGACCCGCTTCCCGTTCGTCCGTCTGTTACTCAAACGAATCAAGGAGGTGTctgtctcttatacacatctccgagccc            >  1:331714/1‑274`  
                                                                                                                                                                                                                                                                                                                      `CACTAAAGGAGACACTATAGGTTTCGACTTCATTACAACAAAAGCGTTAAGAATTTCACGGTTCGCCGTGCTGACCGTTCAATCGTATGTGCGAGCGAGCGCCGAGCTAAGATACCTCTTATTGGTAACACAGTTCCTTTGGCACCGAGCGTCCACATCATTATCACCCGTGGTGACTTTGAGAAAGCAATAGACAAG‑AAACGTCCGGTTCTTAGTGTGGCAGTGACCCGCTTCCAGTTCGTCCGTCTGTTACTCAAACGAACCAAGGAGGTGTTCTGATGGGACTGTTAGATGGTGAAGC            >  1:127281/1‑301`  
                                                                                                                                                                                                                                                                                                                       `aaaAAAGGAGAAAATATAAGTATCGAATGAATTAAAACAAAAGCGTTAAGAATTTAACGGGTCGCCGTGAGGACAGTTCAATAGTATGTGCGAGCGAGGGCTGAGCTAAGAGAACTCGTATTGGTAACACAGTTCCTTTGGCACCGAGAGTCCACAGCATTATCACCCGTGGTGACTTTGAGAAAGGAATAGACAAG‑AAAAGTCCGGTTGTGAGTGTGGAAGTGACAAGCTTCCCGTTCGTCCGTCTGTTACTCAAACGAATCAAGGAGGTGTTCTGATGGGACTGTTAGATGGTGAAGCC           <  1:81896/298‑1`  
                                                                                                                                                                                                                                                                                                                       `aaTAAAGGTGAAACTATATGTTTCGACTTCATTAAAACAAAAGCGTTAAGAATTTCACGGTTCGCCGTGCTGACCGTTCATGCGTAGGTGCGAGCGAGCGCCGAGCTAAGATACCTCTTATTGGTAACACAGTTCCTTTGGAACCGAGCGTCCACATCATTATCACCCGTGGTGACTTTGAGAAAGCAATAGACAAG‑AAACGTCCGGTTCTTAGTGTGGCAGTGACCCGCTTCCCGTTCGTCCGTCTGTTACTCAAACGAATCAAGGAGGTGTTCTGATGGGACTGTTAGATGGTGAAGCC           <  1:1523/299‑1`  
                                                                                                                                                                                                                                                                                                                        `aTAAAGGAGACACTATATGTTTCGACGTCATTACAACAAAAGCGTTAAGAATTTCACGGTTCGCCGTGCTGACCGTTCAATCGTATGTGCGAGCGAGCGCCGAGCTAAGATACCTCTTATTGGTAACACAGTTCCTTTGGCACCGAGCGTCCACATCATTATCACCCGTGGTGACTTTGAGAAAGCAATAGACAAGAAAACGTCCGGTTCTTAGTGTGGCAGTGACCCGCTTCCCGTTCGTCCGTCTGTTACTCAAACGAATCAAGGAGGTGTTCTGATGGGACTGTTAGATGGTGAAGCC           <  1:224912/300‑1`  
                                                                                                                                                                                                                                                                                                                        `CTAAAGGAGACACTATATGTTTCGACTTCATTACAACAAAAGCGTTAAGTATTTCACGGTTCGCCGTGCTGACCGTTCAATCGTATGTGCGAGCGAGCGCCGAGCTAAGATACCTCTTATTGGTAACACAGTTCCTTTGGCACAGAGCGTCCACATCATTATCACCCGTGGTGACTTTGAGAAAGCAATAGACAAG‑AAACGTCCGGTTCTTACTGTGGCAGTGACCCGCTTCCCGTTCGTCCGTCTGTTACTCAAACGAATCAAGGAGGTGATCGGATGTGACTGTTAGATctgtctcttt          >  1:44197/1‑291`  
                                                                                                                                                                                                                                                                                                                        `CTAAAGGAGACACTATATGTTTCGACTTCATTACAACAAAAGCGTTAAGAATTTCACGGTTCGCCGTGCTGACCGTTCAATCGTATGTGCGAGCGAGCGCCGAGCTAAGATACCTCTTATTGGTAACACAGTTCCTTTGTCACCGAGCGTCCACATCATTATCACCCGTGGTGACTTTGAGAAAGCAATAGACAAG‑AAACGTCCGGTTCTTAGTGTGGCAGTGACCCGCTTCCCGTTCGTCCGTCTGTTACTCAAACGAATCAAGGAGGTGTTCTGATGGGACTGTTAGATGGTGAAGCCT          >  1:241274/1‑301`  
                                                                                                                                                                                                                                                                                                                        `CTAAAGGAGACACTATATGTTTCGACTTCATTACAACAAAAGCGTTAAGAATTTCACGGTTCGCCGTGCTGACCGTTCAATCGTATGTGCGAGCGAGCGCCGAGCTAAGATACCTCTTATTGGTAACACAGTTCCTTTGGCACCGAGCGTCCACATCATTATCACCCGTGGTGACTTTGAGAAAGCAATAGACAAG‑AAACGTCCGGTTCTTAGTGTGGCAGTGACCCGCTTCCCGTTTGTCCGTCTGTTACTCAAACGAATCAAGGAGGTGTTCTGATGGGACTGTTAGATGGTGAAGCCc          >  1:143903/1‑300`  
                                                                                                                                                                                                                                                                                                                        `CTAAAGGAGACACTATATGTTTCGACTTCATTACAACAAAAGCGTTAAGAATTTCACGGTTCGCCGTGCTGACCGTTCAATCGTATGTGCGAGCGAGCGCCGAGCTAAGATACCTCTTATTGGTAACACAGTTCCTTTGGCACCGAGCGTCCACATCATTATCACCCGTGGTGACTTTGAGAAAGCAATAGACAAG‑AAACGTCCGGTTCTTAGTGTGGCAGTGACCCGCTTCCCGTTCGTCCGTCTGTTACTCAAACGAATCAAGGAGGTGTTCctgtctcttatacacatctccgagccc          >  1:250421/1‑274`  
                                                                                                                                                                                                                                                                                                                        `CTAAAGGAGACACTATATGTTTCGACTTCATTACAACAAAAGCGTTAAGAATTTCACGGTTCGCCGTGCTGACCGTTCAATCGTATGTGCGAGCGAGCGCCGAGCTAAGATACCTCTTATTGGTAACACAGTTCCTTTGGCACCGAGCGTCCACATCATTATCACCCGTGGTGACTTTGAGAAAGCAATAGACAAG‑AAACGTCCGGTTCTTAGTGTGGCAGTGACCCGCTTCCCGTTCGTCCGTCTGTTACTCAAACGAATCAAGGAGGTGTTCTGATTGGACTGTTAGATGGTGAAGCCT          >  1:31140/1‑301`  
                                                                                                                                                                                                                                                                                                                          `AAAGGAGACACTATATGTTTCGACTTCATTACAACAAAAGCGTTAAGAATTTCACGGTTCGCCGTGCTGACCGTTCAATCGTATGTGCGAGCGAGCGCCGAGCTAAGATACCTCTTATTGGTAACACAGTTCCTTTGGCACCGAGCGTCCACATCATTATCACCCGTGGTGACTTTGAGAAAGCAATAGACAAG‑AAACGTCCGGTTCTTAGTGTGGCAGTGACCCGCTTCCCGTTCGTCCGTCTGTTACTCAAACGAATCAAGGAGGTGTTCTGATGGGACTGTTAGATGGcggtctctta        >  1:205399/1‑291`  
                                                                                                                                                                                                                                                                                                                          `AAAGGAGACACTATATGTTTCGACTTCATTACAACAAAAGCGTTAAGAATTTCACGGTTCGCCGTGCTGACCGTTCAATCGTATGTGCGAGCGAGCGCCGAGCTAAGATACCTCTTATTGGTAACACAGTTCCTTTGGCACCGAGCGTCCACATCATTATCACCCGTGGTGACTTTGAGAAAGCAATAGACAAG‑AAACGTCCGGTTCTTAGTGTGGCAGTGACCCGCTTCCCGTTCGTCCGTCTGTTACTCAAACGAATCAAGGAGGTGTTCTGATGGGACTGTTAGATGGTGAAGCCTGG        >  1:325496/1‑301`  
                                                                                                                                                                                                                                                                                                                          `AAAGGAGACACTATATGTTTCGACTTCATTACAACAAAAGCGTTAAGAATTTCACGGTTCGCCGTGCTGACCGTTCAATCGTATGTGCGAGCGAGCGCCGAGCTAAGATACCTCTTATTGGTAACACAGTTCCTTTGGCACCGAGCGTCCACATCATTATCACCCGTGGTGACTTTGAGAAAGCAATAGACAAG‑AAACGTCCGGTTCTTAGTGTGGCAGTGACCCGCTTCCCGTTCGTCCGTCTGTTACTCAAACGAATCAAGGAGGTGTTCTGATGGGACTGTTAGATGGTGAAGCCTGG        >  1:254616/1‑301`  
                                                                                                                                                                                                                                                                                                                            `AGGAGACACTATATGTTTCGACTTCATTACAACAAAAGCGTTAAGAATTTCACGGTTCGCCGTGCTGACCGTTCAATCGTATGTGCGAGCGAGCGCCGAGCTAAGATACCTCTTATTGGTAACACAGTTCCTTTGGCACCGAGCGTCCACATCATTATCCCCCGTGGTGACTTTGAGAAAGCAATAGACAAG‑AAACGTCCGGTTCTTAGTGTGGCAGTGACCCGCTTCCCGTTCGTCCGTCTGTTACTCAACCGAATCAAGGAGGTGTTCTGATGGGCCTGTTAGATGGTGAAGCCTGGGA      >  1:175230/1‑301`  
                                                                                                                                                                                                                                                                                                                            `AGGAGACACTATATGTTTCGACTTCATTACAACAAAAGCGTTAAGAATTTCACGGTTCGCCGTGCTGACCGTTCAATCGTATGTGCGAGCGAGCGCCGAGCTAAGATACCTCTTATTGGTAACACAGTTCCTTTGGCACCGAGCGTCCACATCATTATCACCCGTGGTGACTTTGAGAAAGCAATAGACAAG‑AAACGTCCGGTTCTTAGTGTGGCAGTGACCCGCTTCCCGTTCGTCCGTCTGTTACTCAAACGAATCAAGGAGGTGTTCTGATGGGACTGTTAGATGGTGAAGCCTGGGA      >  1:128239/1‑301`  
                                                                                                                                                                                                                                                                                                                            `AGGAGACACTATATGTTTCGACTTCATTACAACAAAAGCGTTAAGAATTTCACGGTTCGCCGTGCTGACCGTTCAATCGTATGTGCGAGCGAGCGCCGAGCTAAGATACCCCTTATTGGTAACACAGTTCCTTTTGCACCGAGCGTCCACATCATTATCACCCGTGGTGACTTTGAGAAAGCAATAGACAAG‑AAACGTCCGGTTCTTAGTGTGGCAGTGACCCGCTTCCCGTTCGTCCGTCTGTTACTCAAACGAATCAAGGAGGTGTTCTGATGGGACTGTTAGATGGTGAAGCtgtttt      >  1:230004/1‑295`  
                                                                                                                                                                                                                                                                                                                            `AGGAGACACTATATGTTTCGACTTCATTACAACAAAAGCGTTAAGAATTTCACGGTTCGCCGTGCTGACCGTTCAATCGTATGGGCGAGCGAGCGCCGAGCTAAGATACCTCTTATTTGTAACACAGTTCCTTTGGCACCGAGCGTCCACATCATTATCACCCGTGGTGACTTTGAGAAAGCAATAGACAAG‑AAACGTCCGGTTCTTAGTGTGGCAGTGACCCGCTTCCCGTTCGTCAGTCTGTTACTCAAACGAATCAAGGAGGTGTTCTGATGGGACTGTTAGATGGTGAAGCCTGGGA      <  1:83849/301‑1`  
                                                                                                                                                                                                                                                                                                                             `GGAGACACTATATGTTTCGACTTCATTACAACAAAAGCGTTAAGAATTTCACGGTTCGCCGTGCTGACCGTTCAATCGTATGTGCGAGCGAGCGCCGAGCTAAGATACCTCTTATTGGTAACACAGTTCCTTTGGCACCGAGCGTCCACATCATTATCACCCGTGGTGACTTTGAGAAAGCAATAGACAAG‑AAACGTCCGGTTCTTAGTGTGGCAGTGACCCGCTTCCCGTTCGTCCGTCTGTTACTCAAACGAATCAAGGAGGTGTTCTGATGGGACTGTTAGATGGTGAAGCCctgtct     >  1:6517/1‑295`  
                                                                                                                                                                                                                                                                                                                             `GGAGACACTATATGTTTCGACTTCATTACAACAAAAGCGTTAAGAATTTCACGGTTCGCCGTGCTGACCGTTCAATCGTATGTGCGAGCGAGCGCCGAGCTAAGATACATCTTATTGGTAACACAGTTCCTTTGGCACCGAGCGTCCACATCATTATCACCCGTGGTGACTTTGAGAAAGCAATAGACAAG‑AAACGTCCGGTTCTTAGTGTGGCAGTGACCCGCTTCCCGTTCGTCCGTCTGTTACTCAAACGAATCAAGGAGGTGTTCTGATGGGACTGTTAGATGGTGAAGCCTGGGAA     >  1:52817/1‑301`  
                                                                                                                                                                                                                                                                                                                              `GAGACACTATATGTTTCGACTTCATTACAACAAAAGCGTTAAGAATTTCACGGTTCGCCGTGCTGACCGTTCAATCGTATGTGCGAGCGAGCGCCGAGCTAAGATACCTCTTATTGGTAACACAGTTCCTTTGGCACCGAGCGTCCACATCATTATCACCCGTGGTGACTTTGAGAAAGCAATAGACAAG‑AAACGTCCGGTTCTTAGTGTGGCAGTGACCCGCTTCCCGTTCGTCCGTCTGTTACTCAAACGAATCAAGGAGGTGTTCTGATGGGACTGTTAGATGGctgtctcttataca    >  1:36891/1‑287`  
                                                                                                                                                                                                                                                                                                                               `AGACACTATATGTTTCGACTTCATTACAACAAAAGCGTTAAGAATTTCACGGTTCGCCGTGCTGACCGTTCAATCGTATGTGCGAGCGAGCGCCGAGCTAAGATACCTCTTATTGGTAACACAGTTCCTTTGGCACCGAGCGTCCACATCATTATCACCCGTGGTGACTTTGAGAAAGCAATAGACAAG‑AAACGTCCGGTTCTTAGTGTGGCAGTGACCCGCTTCCCGTTCGTCCGTCTGTTACTCAAACGAATCAAGGAGGTGTTCTGATGGGACTGTTAGATGGTGAAGCCTGGGAAAA   <  1:212877/301‑1`  
                                                                                                                                                                                                                                                                                                                               `AGACACTATATGTTTCGACTTCATTACAACAAAAGCGTTAAGAATTTCACGGTTCGCCGTGCTGACCGTTCAATCGTATGTGCGAGCGAGCGCCGAGCTAAGATACCTCTTATTGGTAACACAGTTCCTTTGGCACCGAGCGTCCACATCATTATCACCCGTGGTGACTTTGAGAAAGCAATAGACAAG‑AAACGTCCGGTTCTTAGTGTGGCAGTGACCCGCTTCCCGTTCGTCCGTCTGTTACTCAAACGAATCAAGGAGGTGTTCTGATGGGACTGTTAGATGGTGAAGCCTGGGAAAA   >  1:210622/1‑301`  

                                                                                                                                                                                                                                                                                                                                                                                                                                                                                                                                                                                                                                                
`TAGCTACATGGAGGAAACACCTGATGGCTCTTTGCGGCACCCATCGTTCGTAATGTTCCGTGGCACCGAGGACAACCCTCAAGAGAAAATGTAATCACACTGGCTCACCTTCGGGTGGGCCTTTCTGCGTTTATAAGGAGACACTTTATGTTTAAGAAGGTTGGTAAATTCCTTGCGGCTTTGGCAGCTATCCTGACGCTTGCGTATATTCTTGCGGTATACCCTCAAGTAGCACTAGTAGTAGTTGGCGCTTGTTACTTAGCGGCAGTGTGTGCTTGCGTGTGGAGTATAGTTAACTGGTAATACGACTCACTAAAGGAG‑‑‑‑‑‑‑‑‑‑‑‑‑‑‑‑‑‑‑‑‑‑‑‑‑‑‑‑‑‑‑‑‑‑‑‑‑‑‑‑‑‑‑‑‑‑‑‑‑‑‑‑‑‑‑‑‑‑‑‑‑‑‑‑‑‑‑‑‑‑‑‑‑‑‑‑‑‑‑‑‑‑‑‑‑‑‑‑‑‑‑‑‑‑‑‑‑‑‑‑‑‑‑‑‑‑‑‑‑‑‑‑‑‑‑‑‑‑‑‑‑‑‑‑‑‑‑‑‑‑‑‑‑‑‑‑‑‑‑‑‑‑‑‑‑‑‑‑‑‑‑‑‑‑‑‑‑‑‑‑‑‑‑‑‑‑‑‑‑‑‑‑‑‑‑‑‑‑‑‑‑‑‑‑‑‑‑‑‑‑‑‑‑‑‑‑‑‑‑‑‑‑‑‑‑‑‑‑‑‑‑‑‑‑‑‑‑‑‑‑‑‑‑‑‑‑‑‑‑‑‑‑‑‑‑‑‑‑‑‑‑‑‑‑‑‑‑‑‑‑‑‑‑‑‑‑‑‑‑‑‑‑‑‑‑‑‑‑‑‑‑‑‑‑‑‑‑‑‑‑‑‑‑‑‑‑‑‑‑‑‑‑‑‑‑‑‑‑‑‑‑  >  NC_001604.1/7461‑7781`  
`‑‑‑‑‑‑‑‑‑‑‑‑‑‑‑‑‑‑‑‑‑‑‑‑‑‑‑‑‑‑‑‑‑‑‑‑‑‑‑‑‑‑‑‑‑‑‑‑‑‑‑‑‑‑‑‑‑‑‑‑‑‑‑‑‑‑‑‑‑‑‑‑‑‑‑‑‑‑‑‑‑‑‑‑‑‑‑‑‑‑‑‑‑‑‑‑‑‑‑‑‑‑‑‑‑‑‑‑‑‑‑‑‑‑‑‑‑‑‑‑‑‑‑‑‑‑‑‑‑‑‑‑‑‑‑‑‑‑‑‑‑‑‑‑‑‑‑‑‑‑‑‑‑‑‑‑‑‑‑‑‑‑‑‑‑‑‑‑‑‑‑‑‑‑‑‑‑‑‑‑‑‑‑‑‑‑‑‑‑‑‑‑‑‑‑‑‑‑‑‑‑‑‑‑‑‑‑‑‑‑‑‑‑‑‑‑‑‑‑‑‑‑‑‑‑‑‑‑‑‑‑‑‑‑‑‑‑‑‑‑‑‑‑‑‑‑‑‑‑‑‑‑‑‑‑‑‑‑‑‑‑‑‑‑‑‑‑‑‑‑‑‑‑‑‑‑‑‑‑‑‑‑‑‑‑‑‑‑‑‑‑‑‑‑‑‑‑‑‑‑taatacgactcactaaaggagACACTATATGTTTCGACTTCATTACAACAAAAGCGTTAAGAATTTCACGGTTCGCCGTGCTGACCGTTCAATCGTATGTGCGAGCGAGCGCCGAGCTAAGATACCTCTTATTGGTAACACAGTTCCTTTGGCACCGAGCGTCCACATCATTATCACCCGTGGTGACTTTGAGAAAGCAATAGACAAG‑AAACGTCCGGTTCTTAGTGTGGCAGTGACCCGCTTCCCGTTCGTCCGTCTGTTACTCAAACGAATCAAGGAGGTGTTCTGATGGGACTGTTAGATGGTGAAGCCTGGGAAAAA  >  NC_001604.1/7899‑8198`

  

Alignment Legend

Aligned base mismatch/match (shaded by quality score): `ATCG/ATCG` `< 3 ≤` `ATCG/ATCG` `< 11 ≤` `ATCG/ATCG` `< 24 ≤` `ATCG/ATCG` `< 35 ≤` `ATCG/ATCG` `< 38 ≤` `ATCG/ATCG`

Unaligned base: `atcg`    Masked matching base: `atcg`    Alignment gap: `‑`    Deleted base: `‑`

Reads not counted as support for junction

read\_name Not counted due to insufficient overlap past the breakpoint.

read\_name Not counted due to not crossing MOB target site duplication.
